# Author Correction: RNAi efficacy is enhanced by chronic dsRNA feeding in pollen beetle

**DOI:** 10.1038/s42003-022-03403-y

**Published:** 2022-05-03

**Authors:** Jonathan Willow, Liina Soonvald, Silva Sulg, Riina Kaasik, Ana Isabel Silva, Clauvis Nji Tizi Taning, Olivier Christiaens, Guy Smagghe, Eve Veromann

**Affiliations:** 1 Institute of Agricultural and Environmental Sciences, Estonian University of Life Sciences, Tartu, Estonia; 2 Department of Plants and Crops, Faculty of Bioscience Engineering, Ghent University, Ghent, Belgium; 3 School of Mental Health and Neuroscience, Faculty of Health, Medicine and Life Sciences, Maastricht University, Maastricht, The Netherlands

**Keywords:** RNAi, Agriculture

Correction to: *Communications Biology* 10.1038/s42003-021-01975-9, published online 06 April 2021.

The wrong Supplementary Data 1 was originally published with this article; it has now been replaced with the correct version. The html version of this article has been corrected.

## Supplementary information

Supplementary Data 1

