# Supplementary material for: RNAi efficacy is enhanced by chronic dsRNA feeding in pollen beetle
Source: Commun Biol. 2021 Apr 6;4:444. doi: 10.1038/s42003-021-01975-9 (PMC8024372; doi:10.1038/s42003-021-01975-9)
Supplement: Supplementary file 2 — Description of Additional Supplementary Files [file 42003_2021_1975_MOESM2_ESM.pdf]

Description of Additional Supplementary Files

File name: Supplementary Data 1

Description: Source data for Fig 1 and Fig 2.
