# Supplementary material for: RNAi efficacy is enhanced by chronic dsRNA feeding in pollen beetle
Source: Commun Biol. 2021 Apr 6;4:444. doi: 10.1038/s42003-021-01975-9 (PMC8024372; doi:10.1038/s42003-021-01975-9)
Supplement: Supplementary file 4 — Reporting Summary [file 42003_2021_1975_MOESM4_ESM.pdf]

nature research

Corresponding author(s): Jonathan Willow and Eve Veromann

Last updated by author(s): Mar 5, 2021

Reporting Summary

Nature Research wishes to improve the reproducibility of the work that we publish. This form provides structure for consistency and transparency in reporting. For further information on Nature Research policies, see our Editorial Policies and the Editorial Policy Checklist.

Statistics

For all statistical analyses, confirm that the following items are present in the figure legend, table legend, main text, or Methods section.

n/a	Confirmed	
☐	☒	The exact sample size (*n*) for each experimental group/condition, given as a discrete number and unit of measurement
☐	☒	A statement on whether measurements were taken from distinct samples or whether the same sample was measured repeatedly
☐	☒	The statistical test(s) used AND whether they are one- or two-sided<br>*Only common tests should be described solely by name; describe more complex techniques in the Methods section.*
☐	☒	A description of all covariates tested
☐	☒	A description of any assumptions or corrections, such as tests of normality and adjustment for multiple comparisons
☐	☒	A full description of the statistical parameters including central tendency (e.g. means) or other basic estimates (e.g. regression coefficient) AND variation (e.g. standard deviation) or associated estimates of uncertainty (e.g. confidence intervals)
☐	☒	For null hypothesis testing, the test statistic (e.g. *F*, *t*, *r*) with confidence intervals, effect sizes, degrees of freedom and *P* value noted<br>*Give P values as exact values whenever suitable.*
☒	☐	For Bayesian analysis, information on the choice of priors and Markov chain Monte Carlo settings
☒	☐	For hierarchical and complex designs, identification of the appropriate level for tests and full reporting of outcomes
☒	☐	Estimates of effect sizes (e.g. Cohen's *d*, Pearson's *r*), indicating how they were calculated

Our web collection on statistics for biologists contains articles on many of the points above.

Software and code

Policy information about availability of computer code

Data collection	No code or software used in data collection
Data analysis	R version 3.6.3

For manuscripts utilizing custom algorithms or software that are central to the research but not yet described in published literature, software must be made available to editors and reviewers. We strongly encourage code deposition in a community repository (e.g. GitHub). See the Nature Research guidelines for submitting code & software for further information.

Data

Policy information about availability of data

All manuscripts must include a data availability statement. This statement should provide the following information, where applicable:

- Accession codes, unique identifiers, or web links for publicly available datasets
- A list of figures that have associated raw data
- A description of any restrictions on data availability

All data are available upon reasonable request.

Field-specific reporting

Life sciences study design

All studies must disclose on these points even when the disclosure is negative.

Sample size	Sample size was determined through making it consistent with similar studies; then we further increased sample size for greater statitical power.
Data exclusions	Data excluded from the study only included insects that died after only one day after beginning the respective experimental replicate. RNAi will not work this quickly, so these were insects were removed from analysis in order to avoid reporting any false positives. This criterion for exclusion was established prior to beginning the study.
Replication	The experiment was replicated three times, each replicate occuring on a different day (three consecutive days).
Randomization	All insects were allocated to their respective treatment in a random manner.
Blinding	Blinding was not possible, as treatment preparation and provision were performed by the same investigators. Furthermore, mortality assessment is a straightforward and nonsubjective endpoint for determination.

Reporting for specific materials, systems and methods

We require information from authors about some types of materials, experimental systems and methods used in many studies. Here, indicate whether each material, system or method listed is relevant to your study. If you are not sure if a list item applies to your research, read the appropriate section before selecting a response.

Materials & experimental systems

n/a	Involved in the study	
☒	☐	Antibodies
☒	☐	Eukaryotic cell lines
☒	☐	Palaeontology and archaeology
☐	☒	Animals and other organisms
☒	☐	Human research participants
☒	☐	Clinical data
☒	☐	Dual use research of concern

Methods

n/a	Involved in the study	
☒	☐	ChIP-seq
☒	☐	Flow cytometry
☒	☐	MRI-based neuroimaging

Animals and other organisms

Policy information about studies involving animals; ARRIVE guidelines recommended for reporting animal research

Laboratory animals	Study did not invlove laboratory animals.
Wild animals	Pollen beetles were caught in the field. Only pollen beetles identified as Brassicogethes aeneus were used in the experiment. All pollen beetles were adults, and very likely of similar age, since they were collected at the same time within the same population. All beetles were caught using a plant-tapping technique, where beetles fell into a funnel leading to a ventilated plastic bottle. Beetles were immediately transported to the laboratory. All unused beetles were released into the natural environment. All surviving beetles in the experiment were euthanised via freezing.
Field-collected samples	Beetles were housed in ventilated plastic containers, and therein provided ad libitum with food and water, in ambient laboratory conditions.During the subsequent three days, three experimental replicates were performed (one replicate per day). After setup for each replicate, experimental pollen beetles were immediately placed under controlled conditions in a climate chamber, at 20 degrees C, 60% relative humidity and a light:dark cycle of 16:8 hours.. End-of-experiment protocol involved euthanising all surviving experimental beetles via freezing.
Ethics oversight	No ethical approval or guidance was required for the study. Pollen beetles are common crop pests, are not legally protected, and require no ethical approval regarding their handling. Nevertheless, all handlings with these insects were performed ethically. Guidance of other aspects of the study took place through consistancy with other studies within this field of research, especially those involving pollen beetles.

Note that full information on the approval of the study protocol must also be provided in the manuscript.
